# Management of a huge symptomatic hemorrhagic mesenteric cyst

**DOI:** 10.1002/ccr3.3299

**Published:** 2020-09-15

**Authors:** Alfred Ogwal, Herbert Ariaka, Vincent Medeyi, Emmanuel Nkonge, Felix Oyania

**Affiliations:** ^1^ St. John of God Hospital Tigania Kenya; ^2^ Uganda Heart Institute Kampala Uganda; ^3^ Salem Kolonyi Hospital Mbale Uganda; ^4^ Kitovu Hospital Masaka Uganda; ^5^ Paediatric Surgery Mbarara National Referral Hospital Mbarara Uganda

**Keywords:** acute, conservative, cyst, elective, mesenteric

## Abstract

Mesenteric cysts are uncommon benign tumors with atypical clinical presentations. An acute presentation may not necessarily warrant emergency surgery, and planned surgery can achieve excellent results if the patient is hemodynamically stable at presentation.

## Graphical abstract

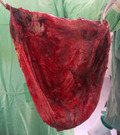

## 1 INTRODUCTION

Mesenteric cysts are rare benign intra‐abdominal tumors that are often misdiagnosed because of their varied clinical presentations. This report describes the case of a patient who presented acutely. The patient was initially managed conservatively and later surgically. Open laparotomy was performed; histology revealed a benign hemorrhagic cyst of the mesentery.

Mesenteric cysts are uncommon benign intra‐abdominal tumors that occur across the life course, with a reported incidence of 1/100 000 in adults and 1/20 000 in children and a female‐to‐male ratio of 2:1.^1^ Lesions are typically located in the small bowel mesentery (66%) or large bowel mesentery (33%),^2^ with occasional extension to the retroperitoneum.^3^ Depending on their size and location, these cysts are often asymptomatic and are thus discovered incidentally during routine imaging; symptomatic cases may present with acute or chronic vague abdominal pain (55%‐81%), a palpable mass (44%‐61%), abdominal distension (17%‐61%), nausea and vomiting (45%), constipation (27%), or diarrhea (6%).^1^ In the worst‐case scenario, there may be complete bowel obstruction, obstructive uropathy, peritonitis (from hemorrhagic or infected cysts), or volvulus.^4^

Complete surgical excision, by either open or laparoscopic approach, ensures the lowest risk of recurrence. This report highlights the case of a 47‐year‐old patient who presented acutely but was successfully managed semi‐electively.

## 2 CASE PRESENTATION

### 2.1 Patient information

A 47‐year‐old farm worker presented to our hospital with a 1‐year history of progressive abdominal distension that was initially painless but became painful 3 days prior to admission. At the time of presentation, he reported falling on his abdomen while working with cattle in the field 3 days earlier. He also reported a history of difficulty in breathing while lying supine. However, there was no history of cough. The patient had normal bowel and bladder habits.

### 2.2 Clinical findings

Physical examination revealed the patient to be in fair general condition with no wasting. He was not febrile, anemic, or jaundiced. There was great abdominal distension with no clear delineation of any abdominal masses. Other systemic examinations were unremarkable, with no clinically significant findings.

### 2.3 Diagnostic assessments

The initial blood work‐up included complete blood count, liver function, renal function, and serum electrolytes, all of which were within normal ranges. The patient was hepatitis B surface antigen‐negative and sero‐negative for human immunodeficiency virus.

**Figure 1:**
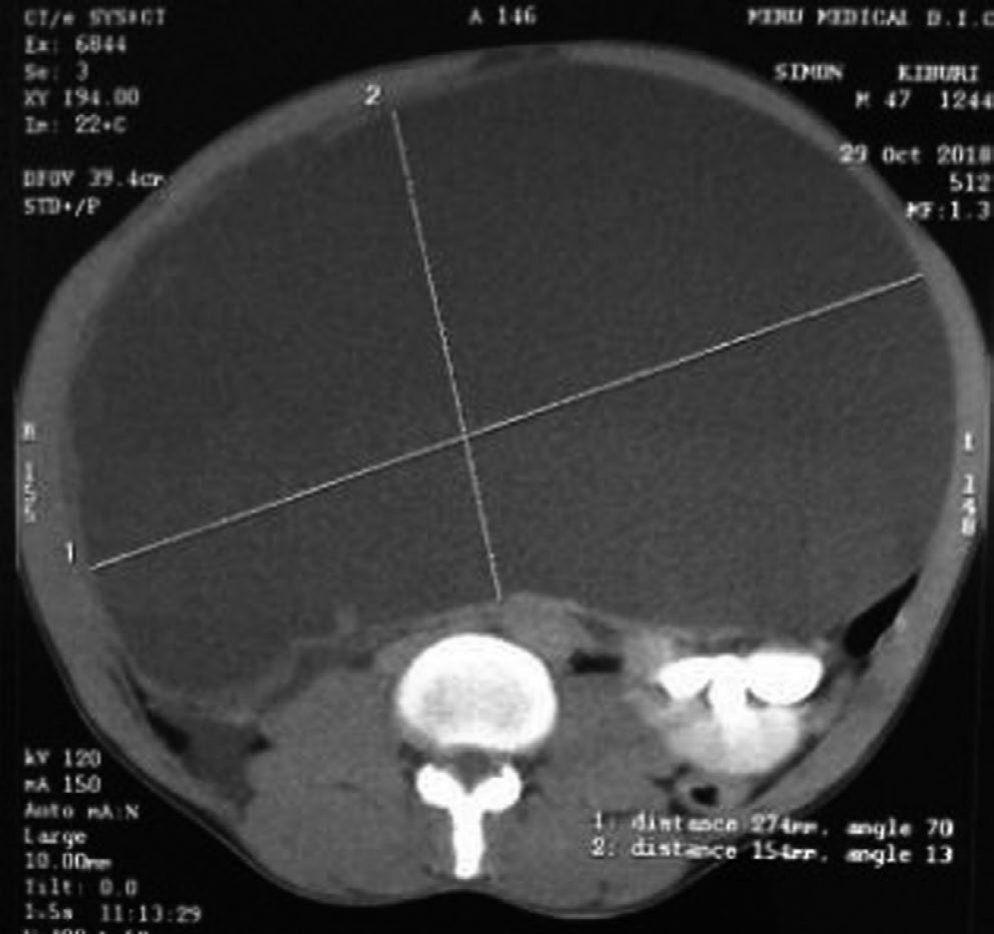
Abdominal computed tomography scan showing the extent of the tumor, coronal view

Imaging investigations included an abdominopelvic ultrasound scan, which revealed a cystic mass occupying much of the abdominal cavity, with no clear site of origin. A contrast‐enhanced computed tomography (CT) scan of the abdomen showed a cystic mass extending from the lower edge of the pancreatic tail to the pelvic brim, with anterior and posterior extension almost to the kidneys. The cyst measured approximately 15.4 cm X 27.4 cm (Figure 1).

The above features suggested the presence of a large mesenteric cyst, with a differential diagnosis of a large pancreatic pseudocyst. On the basis of the presentation and imaging findings, the patient was prepared for exploratory laparotomy within 1 week.

### 2.4 Therapeutic intervention

**Figure 2:**
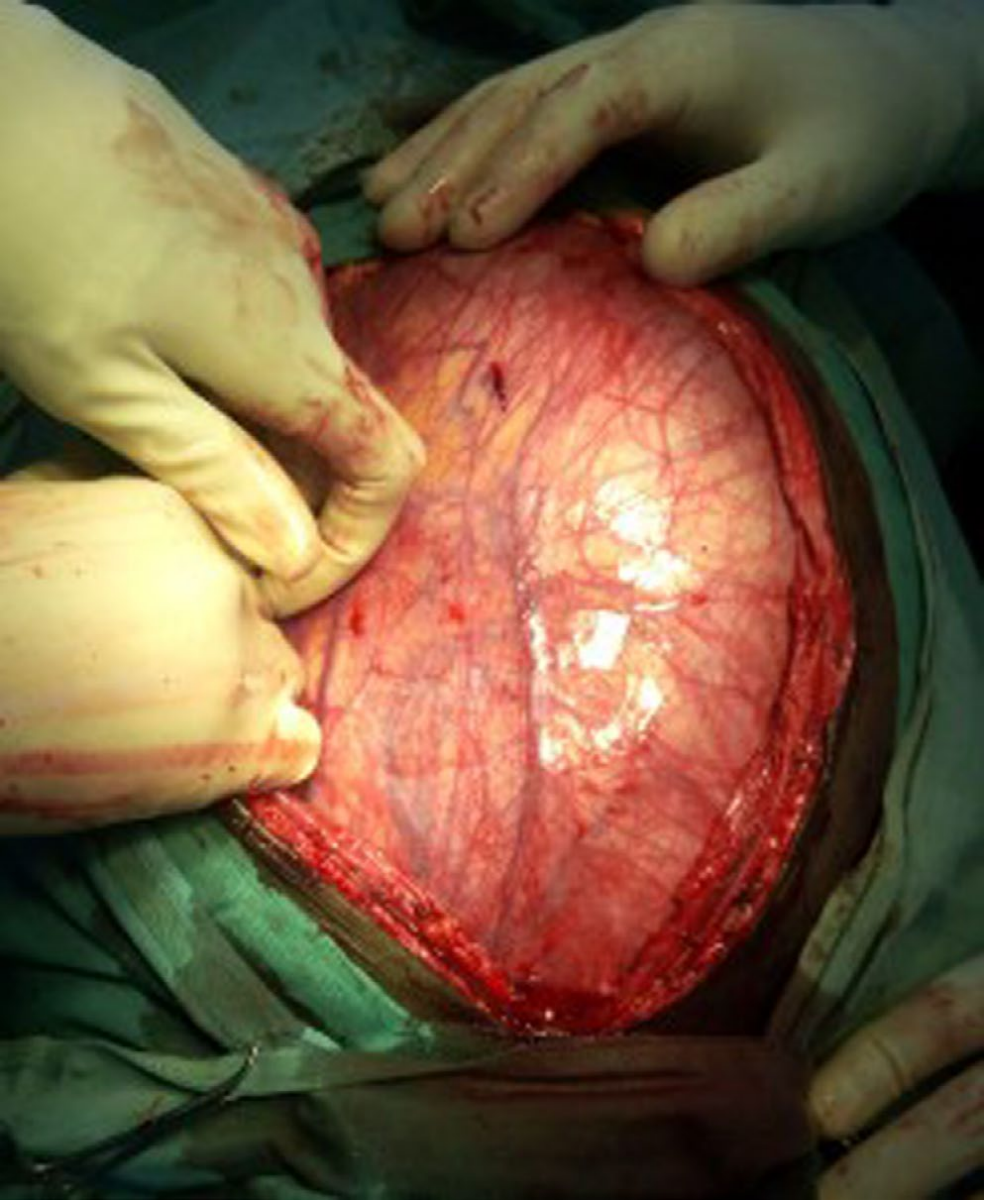
Cyst occupying the entire abdominal cavity immediately after laparotomy incision, anterior view

**Figure 3:**
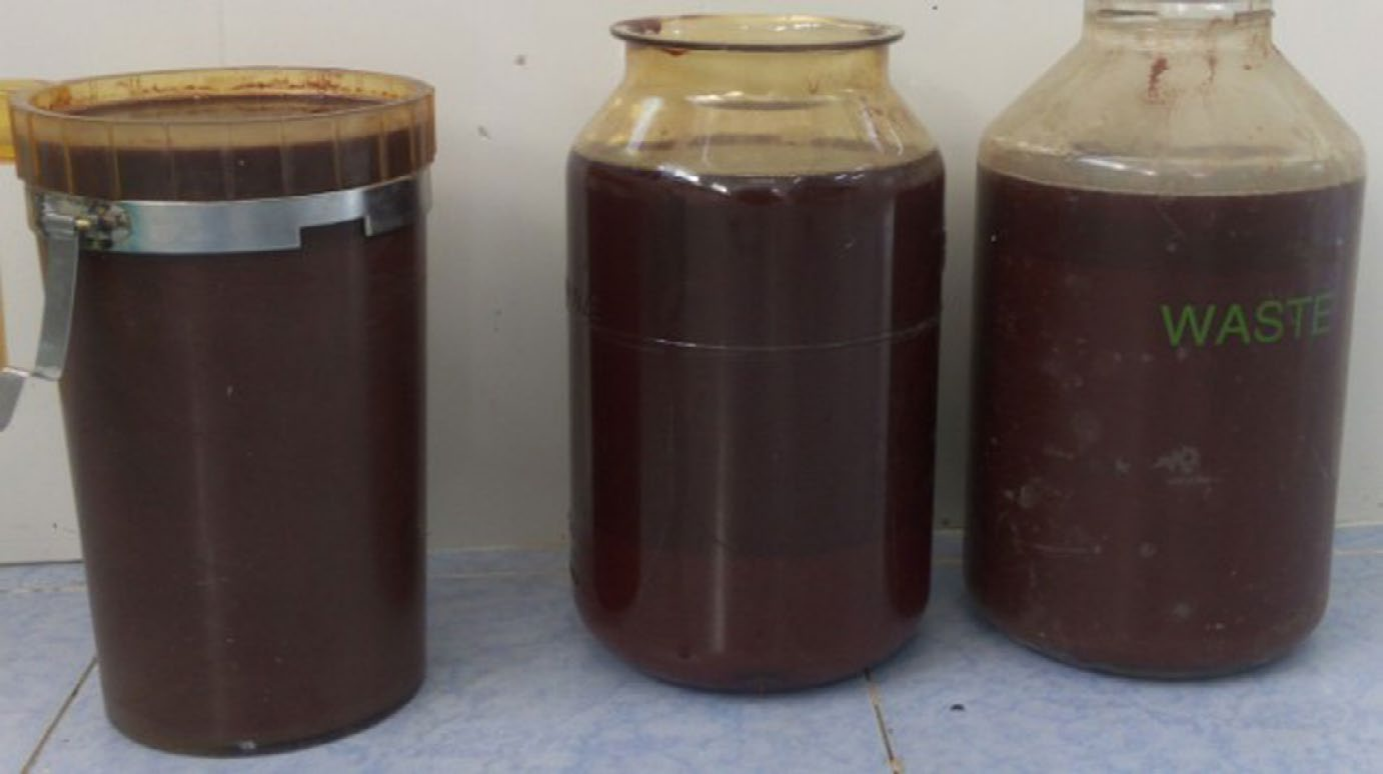
Containers filled with the approximately 8 L of cyst fluid that was drained prior to the dissection of the cyst wall from the bowel mesentery

Initial management included analgesia and broad‐spectrum antibiotics for symptom relief and for treatment of a suspected infected cyst. Because of the suspicion of infection, exploratory surgery was delayed. After 1 week, the decision was made to perform exploratory laparotomy. When the abdomen was opened, the cyst was observed to occupy much of the abdominal cavity (Figure 2). The cyst fluid was drained to facilitate the dissection (Figure 3).

**Figure 4:**
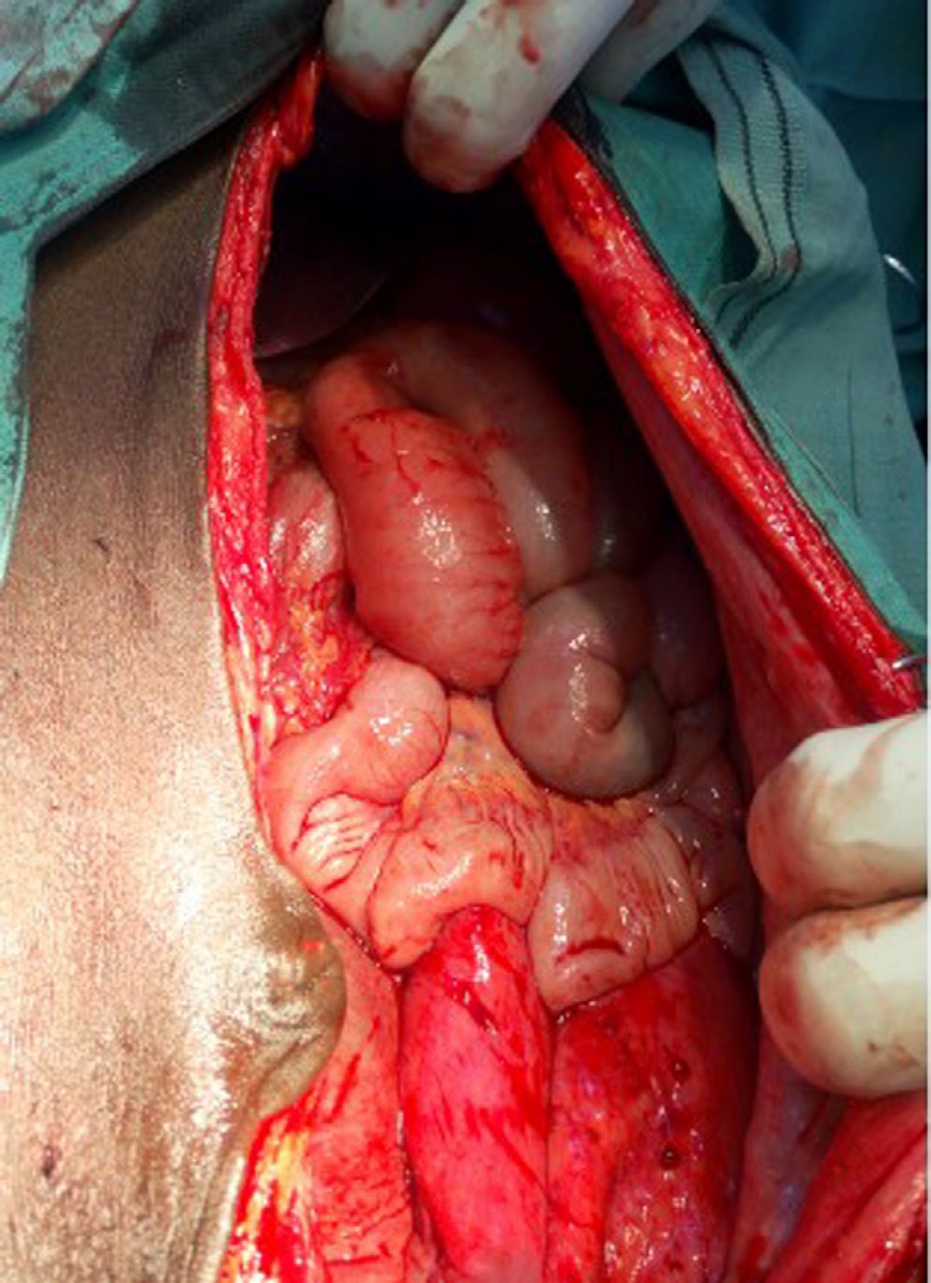
Abdominal cavity after complete excision of the cyst

**Figure 5:**
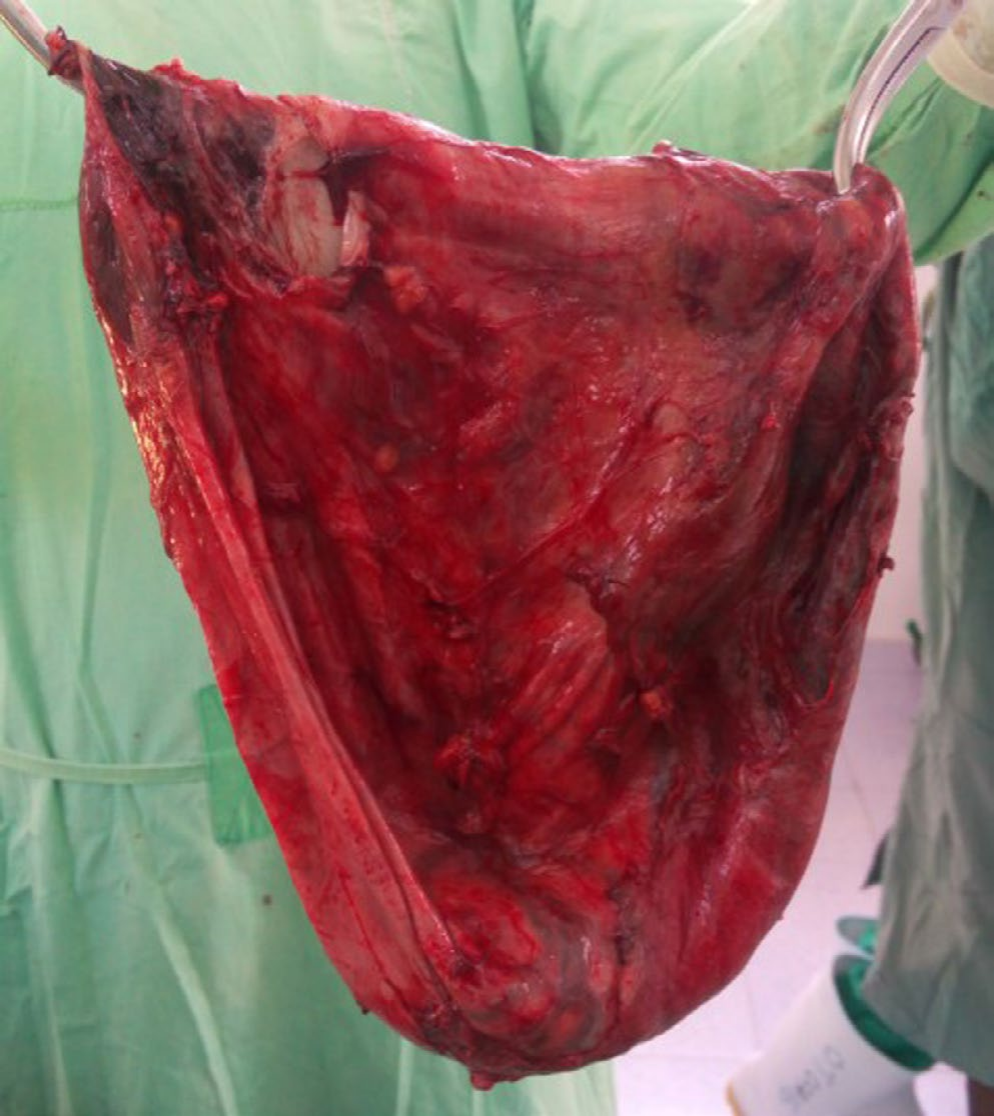
Completely excised cyst

Close inspection revealed involvement of the mesentery of the ileum, with the appendix firmly adhered to the cyst wall. No adhesions were found between the cyst wall and other intra‐abdominal structures. The cyst was carefully dissected from the mesentery of the ileum, sparing the blood supply. Complete enucleation of the cyst was accomplished without the need for bowel resection. However, appendectomy was performed because the appendix was firmly adhered to the cyst wall (Figures 4 and 5).

The final specimen sent for histopathology was a thick‐walled disk‐shaped cystic mass measuring 20 × 18 × 40 cm. The immediate postoperative period was uneventful, and the patient was discharged on the third day after the surgery.

### 2.5 Follow‐up and outcomes

By the end of the first week postsurgery, there was complete healing of the surgical incision with no wound infection. Histology confirmed a benign hemorrhagic cyst of the mesentery, with the appendix attached to the wall of the cyst. Subsequent reviews were conducted every 2 weeks for 1 month and then at 3 months and again at 6 months. Follow‐up abdominal ultrasound scans revealed no suspicious intra‐abdominal lesions.

## 3 DISCUSSION

Mesenteric cysts are rare intra‐abdominal tumors with varied presentations ranging from incidental discovery during routine imaging investigations to diagnosis following complications such as bowel obstruction.^5^, ^6^

The patient described in this report presented with a long‐standing history of progressive abdominal distension, 3 days of abdominal pain, and a recent fall on the abdomen. The abdominal pain may have been caused by probable hemorrhage into the cyst resulting from a trivial trauma to the abdomen. It is also possible that the abdominal pain was caused by infected cyst fluid, but this is unlikely because the patient was afebrile and because his complete blood count was within the normal range.

The etiology of cysts of this type is unclear. Some theories suggest a benign proliferation of ectopic mesenteric tissue that fails to communicate with the core lymphatic system.^7^ However, these cysts may result from a number of etiologies, including previous pelvic surgery (as was observed in a case presented by Jain et al^5^), trauma, inflammatory disease, endometriosis, and neoplasia.

Imaging such as ultrasound scan, CT scan, and magnetic resonance imaging are the investigation modalities of choice. In the case presented here, a CT scan was sufficient to have a high index of suspicion of mesenteric cyst, although a huge pancreatic pseudocyst was considered as a differential diagnosis.

Approaches to the management of mesenteric cysts vary in the available literature. Surgical excision, either by laparotomy or laparoscopy, is the preferred approach.^8^, ^9^, ^10^ Laparoscopic resection can be attempted for smaller tumors, but laparotomy was preferred in the present case, given the size of the patient's cyst. Acute surgical resection has been the dominant approach for symptomatic mesenteric cysts. However, Leung et al have demonstrated that, in acute settings, conservative management followed by elective surgery is also a safe approach.^1^ In our case, the patient's main concern of abdominal pain was managed conservatively with analgesics and broad‐spectrum antibiotics, and laparotomy was performed 1 week later.

Emergency surgery has inherent risks of complications such as bowel perforation, damage to surrounding structures, wound infection, and intra‐abdominal abscess. In the case presented here, performing the surgery semi‐electively avoided these complications associated with emergency surgery. This approach also ensured that the surgical team had time to prepare mentally and in terms of the necessary skills because this was the first case of its kind to be handled in our facility. However, such an approach is only possible if the patient is stable.

## 4 CONCLUSION

Mesenteric cysts presenting in acute settings can initially be managed conservatively, followed by a planned elective surgical excision of the cyst, preferably by laparotomy. However, the decision to delay surgery should be considered on an individual basis. Overall, the outcome from this approach is excellent if the proper decision is made, as our case clearly demonstrates.

## CONFLICT OF INTEREST

None declared.

## AUTHOR CONTRIBUTIONS

AO: was a major contributor in writing the manuscript and was the lead surgeon in the management of the patient. HA and VM: participated in the literature review and in revising the manuscript. FO and EN: were involved in critically revising the manuscript for important intellectual content. All authors: read and approved the final manuscript.

## ETHICAL APPROVAL

Written informed consent was obtained from the patient for publication of this report and any accompanying images. A copy of the consent form is available for review by the Editor‐in‐Chief of this journal.
